# Rolling Motion Along an Incline: Visual Sensitivity to the Relation Between Acceleration and Slope

**DOI:** 10.3389/fnins.2018.00406

**Published:** 2018-06-22

**Authors:** Francesca Ceccarelli, Barbara La Scaleia, Marta Russo, Benedetta Cesqui, Silvio Gravano, Maura Mezzetti, Alessandro Moscatelli, Andrea d’Avella, Francesco Lacquaniti, Myrka Zago

**Affiliations:** ^1^Centre of Space Bio-Medicine, University of Rome Tor Vergata, Rome, Italy; ^2^Laboratory of Neuromotor Physiology, IRCCS Santa Lucia Foundation, Rome, Italy; ^3^Department of Economics and Finance, University of Rome Tor Vergata, Rome, Italy; ^4^Department of Systems Medicine, University of Rome Tor Vergata, Rome, Italy; ^5^Department of Biomedical and Dental Sciences and Morphofunctional Imaging, University of Messina, Messina, Italy

**Keywords:** gravity, internal models, Bayesian, virtual reality, mental simulations

## Abstract

People easily intercept a ball rolling down an incline, despite its acceleration varies with the slope in a complex manner. Apparently, however, they are poor at detecting anomalies when asked to judge artificial animations of descending motion. Since the perceptual deficiencies have been reported in studies involving a limited visual context, here we tested the hypothesis that judgments of naturalness of rolling motion are consistent with physics when the visual scene incorporates sufficient cues about environmental reference and metric scale, roughly comparable to those present when intercepting a ball. Participants viewed a sphere rolling down an incline located in the median sagittal plane, presented in 3D wide-field virtual reality. In different experiments, either the slope of the plane or the sphere acceleration were changed in arbitrary combinations, resulting in a kinematics that was either consistent or inconsistent with physics. In Experiment 1 (slope adjustment), participants were asked to modify the slope angle until the resulting motion looked natural for a given ball acceleration. In Experiment 2 (acceleration adjustment), instead, they were asked to modify the acceleration until the motion on a given slope looked natural. No feedback about performance was provided. For both experiments, we found that participants were rather accurate at finding the match between slope angle and ball acceleration congruent with physics, but there was a systematic effect of the initial conditions: accuracy was higher when the participants started the exploration from the combination of slope and acceleration corresponding to the congruent conditions than when they started far away from the congruent conditions. In Experiment 3, participants modified the slope angle based on an adaptive staircase, but the target never coincided with the starting condition. Here we found a generally accurate performance, irrespective of the target slope. We suggest that, provided the visual scene includes sufficient cues about environmental reference and metric scale, joint processing of slope and acceleration may facilitate the detection of natural motion. Perception of rolling motion may rely on the kind of approximate, probabilistic simulations of Newtonian mechanics that have previously been called into play to explain complex inferences in rich visual scenes.

## Introduction

There is no question that, when it comes to acting on a visible falling object, people normally anticipate gravity and inertia effects quite accurately (Lee et al., 1983; Lacquaniti and Maioli, 1989; Michaels et al., 2001; Zago et al., 2004). A case in point is represented by the manual interception of a ball rolling down an inclined plane with an acceleration that varies with a trigonometric function of the incline angle (Galileo’s law, Galilei, 1638). Even 3-years old children can be successful at such a task (Rosey et al., 2008), and adults can easily deal with many different accelerations of a descending target, as resulting from variable combinations of incline angles and initial target positions (Tresilian and Lonergan, 2002; La Scaleia et al., 2014, 2015).

It is therefore puzzling that human observers asked to judge the artificial animation of a target descending along an incline are generally poor at detecting motion anomalies. Bozzi (1959, 1961) projected a square target sliding down a plane on a screen, and asked observers to choose the motion function, among several alternatives, which looked like the most natural, frictionless motion. He found that sliding is perceived as most natural when the target accelerates at the start and then moves at constant speed, instead of when it is uniformly accelerated as expected from physics. Hecht (1993) used computer-generated displays of wheels rolling down an inclined plane. His participants reported that the displays looked equally natural under very different motion laws; they were unable to differentiate between different acceleration functions by detecting the specific effects of gravity. Moreover, their judgments were based mainly on the translation component of the rolling motion, while rotation tended to be neglected (see also Vicario and Bressan, 1990). Rohrer (2003), instead, asked participants to rate the naturalness of computer animations depicting a circular marble rolling down curved slopes with kinematics conforming either to Newtonian mechanics or to a naïve belief violating gravity constraints (Rohrer, 2002). He found that participants rated the incorrect version as more natural than the correct one.

But how can actions be so accurate if the eliciting target motions are so misperceived? This kind of dissociation is often accounted for by invoking the so called dual-system hypothesis (e.g., Zago and Lacquaniti, 2005), according to which visual information for action and visual information for perception involve different processes, possibly mediated by different cerebral networks (Goodale and Milner, 1992; Jeannerod et al., 1995; Tresilian, 1995). However, one should also consider a much simpler explanation -not necessarily alternative to the dual-system hypothesis- to account for the apparent discrepancy of the results summarized above, namely that the visual cues and context involved in the perceptual experiments were drastically different from those of the motor experiments. In fact, the accurate interception of a ball rolling down an incline involved real, familiar objects viewed under rich, naturalistic conditions (La Scaleia et al., 2014, 2015), whereas the inaccurate perceptions of descending motions involved abstract, unfamiliar objects, presented with limited context and impoverished stimuli (Bozzi, 1959; Hecht, 1993; Rohrer, 2003).

Motor interceptions can still be accurate even when the free-falling target is virtual, but only if the visual scene is rich of contextual cues providing a correct environmental reference and scale, whereas the success rate degrades considerably when the target is embedded in a blank scene (Miller et al., 2008) or in a scene with an incongruent reference to gravity (Zago et al., 2011). Similar conclusions were drawn from a perceptual task involving the visual discrimination of motion duration of targets moving in different directions (Moscatelli and Lacquaniti, 2011).

The experimental evidence that a ball rolling down an incline can be easily intercepted (La Scaleia et al., 2014, 2015) demonstrates the availability of adequate neural information about the specific coupling between target kinematics and incline angle, the issue being the context under which this information becomes accessible also in perceptual tasks, independently of a motor action. In line of principle, virtual reality may provide such a context (e.g., Carrozzo and Lacquaniti, 1998; Keshner, 2004). On the one hand, it allows the display of quasi-realistic scenes with the kind of visual cues (stereo, familiar size, perspective, shading, texture gradient, lighting, etc.) that have been shown to be critical for predictions of visual target motion (Senot et al., 2005; Diaz et al., 2013), hand interception of projectiles accelerated by gravity (Russo et al., 2017), and weight perception from motion on a slope (Zintus-art et al., 2016). On the other hand, it allows the independent experimental manipulation of incline slope and target acceleration.

Here we used immersive 3D virtual reality with a wide field of view (FOV) to present a sphere rolling down an inclined plane located in the median sagittal plane. Stimuli were shown stereoscopically in the near space (within about 2 m from the observer), since it is known that perceptual estimates of slant (plane inclination relative to the horizontal) tend to be more accurate in the near (peri-personal) space than in the far space, and when stereoscopic cues are available (Bridgeman and Hoover, 2008; Li and Durgin, 2010; Hecht et al., 2014). In different experiments, either the slope of the plane or the sphere acceleration could be changed in arbitrary combinations, resulting in a kinematics that was either consistent or inconsistent with physics. In Experiment 1 (slope adjustment), participants were asked to modify the slope angle until the resulting motion looked natural for a given ball acceleration. In Experiment 2 (acceleration adjustment), instead, they were asked to modify the acceleration until the motion on a given slope looked natural. We tested both slope adjustments and acceleration adjustments to probe separately the use of these two cues in the assessment of naturalness of descending motion along an incline.

In these two experiments, we found that participants were rather accurate at finding the correct match between slope angle and ball acceleration, but there was a systematic effect of the initial conditions: accuracy was higher when the participants started the exploration from the combination of tilt and acceleration corresponding to the natural conditions than when they started far away from the natural conditions. To control for the effect of the initial conditions, we carried out a third experiment involving an adaptive staircase to modify the slope (slope adjustment as in Experiment 1), but avoiding the coincidence of starting condition and target, and we found a generally accurate performance.

## Experiment 1

In this experiment we asked participants to find the slope angle that matched the observed acceleration of the rolling ball (slope adjustment). In each trial, the ball rolled down with a preset acceleration consistent with a theoretical tilt of the plane of 19°, 39°, or 60°, which was generally different from the actual tilt of the plane that was shown initially. Participants adjusted iteratively the tilt of the visible plane within the range 6°/70°, until they judged that the ball motion on the newly found inclination looked the most natural among all tested motions.

### Methods

#### Participants

Fifteen subjects (10 females and 5 males, 30.4 ± 7.2 years old, mean ± SD) participated in Experiment 1. All participants in this and the following experiments were right-handed (as assessed by a short questionnaire based on the Edinburgh scale), had normal or corrected-to-normal vision, no past history of psychiatric or neurological diseases, and were naïve to the specific purpose of the experiments. They gave written informed consent to procedures approved by the Institutional Review Board of Santa Lucia Foundation (protocol n. CE/PROG.454), in conformity with the Declaration of Helsinki on the use of human subjects in research. Sample size (*n* = 15) was determined *a priori* based on previous studies from our laboratory involving motor protocols with an inclined plane (La Scaleia et al., 2014, 2015), and on the effects observed in the participants (different from those of the present experiments) of a pilot study.

#### Apparatus and Visual Stimuli

**FIGURE 1:**
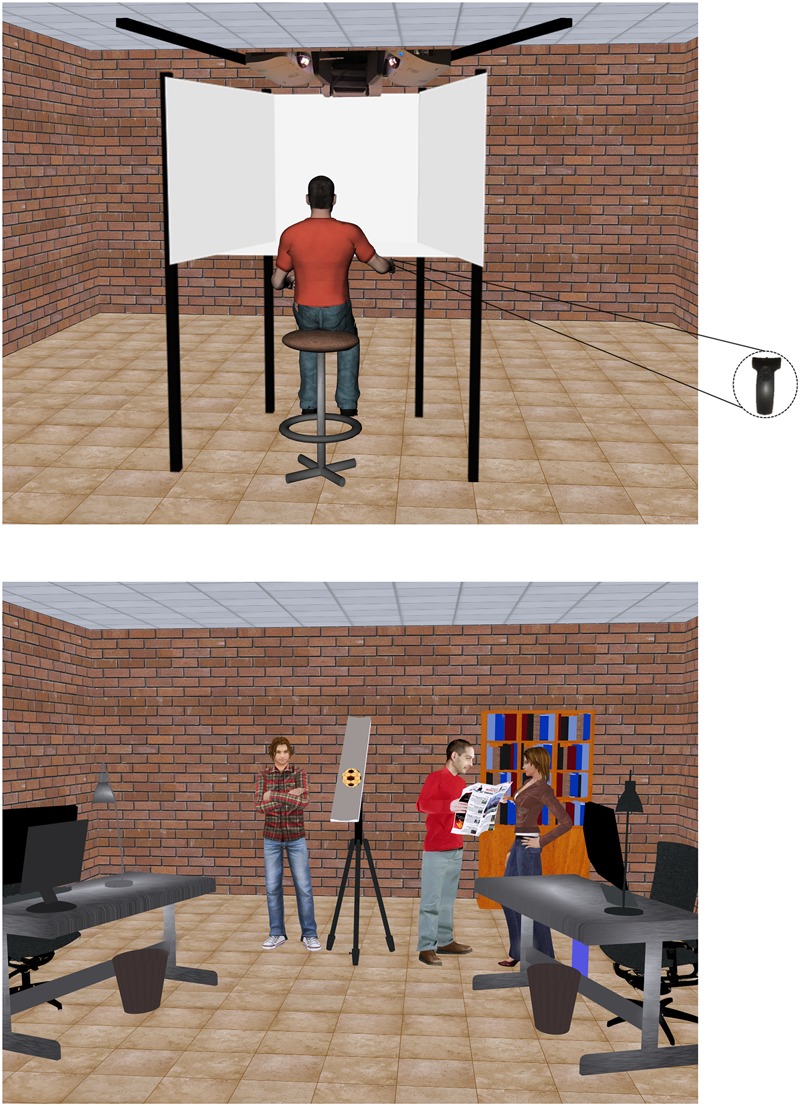
Experimental set-up and 3D visual scenario.

The participants sat on a height-adjustable chair in front of a mini CAVE (Cave Automatic Virtual Environment) in a dark room. They wore shutter glasses and held a 6DOF Wand Sensor (IS-900 system, InterSense Inc., Bedford, MA, United States) in the right hand (see **Figure 1**). The mini CAVE (VRMedia S.r.l., Pisa, Italy) consisted of four projection screens: a frontal screen 1.05 m wide and 1.05 high, two lateral screens 1.40 m wide and 1.05 m high, which were tilted by 15°23′ relative to the sagittal plane (to the left or right for the left and right screen, respectively), and a horizontal screen of trapezoidal shape (isosceles trapezoid) with 0.98 m height and bases length of 1.05 and 1.57 m (for the near and far side relative to the observer, respectively). All CAVE walls were front-projection screens and the optic paths were halved by means of mirrors. Position and height of the chair were adjusted so that the eyes of each participant were located at a horizontal distance of about 0.95 m from the frontal screen and roughly centered on the frontal screen midpoint. The horizontal and vertical FOV were about 180° and 160° respectively. 3D visual scenario and stimuli were generated with XVR (eXtreme Virtual Reality, VRMedia S.r.l., Pisa, Italy, Tecchia et al., 2010), and were rendered in quasi-real time by an HP workstation Z210 with an ATI Firepro 3D V7900 graphics card (master PC). Two slaves HP workstations Z210 drove synchronously the 3D rendered graphical output to 4 LCD front projectors for screen display (3 NEC U300XG for the left, right, and frontal screen, ACER S5301WM DLP 3D-ready for the horizontal screen; 60-Hz stereo frame rate; 1024 × 768 pixels resolution for the left, right, and frontal screen, and 1280 × 800 pixels for the horizontal screen). Head position and orientation were tracked on-line by means of an inertia-ultrasound motion tracking system Intersense IS 900 (IS-900 system, InterSense Inc., Bedford, MA, United States). A 6DoF Intersense sensor was placed on top of the bridge of the stereo shutter glasses (OPTOMA 3D-RF, Optoma Europe Ltd., Watford, United Kingdom) to update the virtual scene based on head position and orientation. In separate tests, we measured an average update latency of 3 stereo frames. Button-press time of Wand Sensor and coordinates of Intersense sensor on the shutter glasses were sampled by XVR at 1 kHz.

The scene background depicted part of a furnished laboratory (4 m wide, 7 m long, 3.10 m high in world scale), as a realistic version of the actual laboratory where the experiments were performed. The scene was projected at a 1:1 scale, with truthful width-depth rendering. Three human characters imported from Autodesk 3ds Max 2010 were placed in different locations of the virtual laboratory to provide additional cues about the scene reference and approximate metric scale. All elements of the background scene, including the human characters, were static. Perspective geometry, textures, directional lights, and shadows were included in the scene to augment 3D effects. Overhead lighting was provided.

An inclined plane (1.8 m long, 0.17 m wide) supported by a tripod was placed 30 cm to the left of the center line of the background wall, with the longitudinal axis orthogonal to this wall. The plane could be tilted relative to the horizontal by an angle specified by the protocol (see below). A textured ball (diameter, 13.5 cm), initially at rest over the plane, was released and rolled down with an acceleration specified by the protocol. The pivoting point for tilting the plane corresponded to the position of the ball center at the time when the ball fell off the plane (exit point). Irrespective of the tilt angle, the height of the ball at the exit point was 1.05 m above the floor. Notice that the inhomogeneous texture of the ball surface provided optical cues about the rotational component of the motion. The position of the observer’s head in front of the scene was such that the eyes were centered on the longitudinal axis of the plane, 30 cm above and at 1.14 m horizontal distance from the apparent position of the lower end of the plane (see **Figure 4**). The room lights were extinguished and extraneous light sources eliminated with the result that the room and apparatus were poorly visible with the stimuli displayed.

#### Ball Kinematics

Starting from rest, the ball rolled down the plane without slipping or bouncing^1^. The simulated motion corresponded to that of a sphere with a homogenous mass distribution, accelerated by Earth gravity (*g* = 9.81 m/s^2^) and with negligible rolling resistance. Motion equation was:

(1) $$s=\frac{1}{2}\frac{5}{7}g(\sin\;\theta_{b})t^{2}$$

where *s* is the time-varying position of the center of mass of the ball along the plane axis, and *𝜃_b_* is the plane tilt relative to the horizontal. The linear acceleration a of the ball center of mass was:

(2) $$a=\frac{5}{7}gsin\theta_{b}$$

In Equations 1 and 2, the subscript *b* in *𝜃_b_* stands for *ball*, to underline the fact that the tilt angle of the plane that was congruent with the displayed kinematics of the ball could be different from the tilt of the plane *𝜃_i_* that was actually shown each time; in other words, *𝜃_b_* was a hidden variable (see below). The angular speed (magnitude of the angular velocity vector) of ball rotation around its center of mass by definition corresponds to the ratio between the instantaneous speed of the center of mass and the radius of the ball. Once the ball reached the lower end of the plane, it fell off with parabolic motion, but this segment of the animation was poorly visible due to the screen geometry.

#### Instructions to the Participants

Before the experiment, we provided the following written instructions (in Italian): “Upon pressing the *Start* button of the Wand, the ball at rest over the plane will start rolling down and will eventually fall to the floor, disappearing from the visual scene. Next, you will see again the ball at rest over the plane and in the same initial position as before. Your task is to change the plane tilt until ball motion looks natural to you. At any time after ball reappearance at rest, you can change the tilt of the plane by pressing either the *Increase* or the *Decrease* button of the Wand, or you can leave the current tilt by pressing the *Same* button. *Increase* and *Decrease* buttons will increase and decrease the tilt by 1°, respectively. These buttons will be effective within the allowed range of possible tilts. Press again the *Start* button to watch the ball motion on the plane. Beware that the task may require several changes, and you may need to switch repeatedly between *Increase, Decrease, Same*, and *Start* buttons. Press the *OK* button when the ball motion appears to you as the most natural of all tested motions. The plane will then disappear to reappear again after a short delay with the ball at rest. To begin the new trial, press the *Start* button. All trials are similar, except for the fact that the initial position of the ball and/or the plane tilt will be different. Whenever you wish to pause, simply refrain from pressing the *Start* button.”

#### Procedures

After the instructions, participants familiarized with the virtual environment of the mini CAVE. All of them reported correct vision in the 3D environment by confirming that they saw the ball in 3D (instead of seeing two different images). The experimenter performed 5 trials to demonstrate the general protocol. In these trials, the stimuli were unrelated to the experimental ones (different initial plane tilts and accelerations), and the experimenter provided random responses not to provide any information about the criterion for choosing one or another plane tilt. The experiment started immediately afterward. The plane was initially shown at one of 3 different tilt angles *𝜃_i,s_*: 19°, 39°, or 60°. After a pseudorandom delay between 250 and 550 ms (in 100 ms steps) from the *Start* button press, the ball rolled down the plane from a given initial position *s_i_*, with the law of motion described by Equations 1 and 2 and with a value of *𝜃_b_* corresponding to 19°, 39°, or 60°, depending on the trial. As explained in the instructions, participants could adjust iteratively the actual tilt of the plane *𝜃_i_* within the range 6°/70° in 1° steps, starting from the initial value *𝜃_i,s_*. When the value of *𝜃_i_* set by the participant fell outside the preset range, the computer program replace it with the immediately preceding value. The participant’s response corresponded to the plane tilt *𝜃_i_* that was judged as the most natural (i.e., when the participant pressed the *OK* button), and it was stored together with the preceding sequence of changes. If the participant tried to go beyond the allowed range in 3 consecutive attempts, the trial was terminated and the last value of *𝜃_i_* was taken as the response. Notice that the participants were not prevented from choosing the first presented stimulus as natural, without any adjustment of the plane tilt and without any re-observation of ball motion. These responses will be denoted *immediate responses* in the following. No feedback was provided to the participants about either their responses, the number of steps in the sequence or the condition they were currently exploring. On average, reaching a final decision required 23 adjustments, and the average duration of the experimental session was 2 h and 22 min (including the pauses).

#### Protocol Design

**FIGURE 2:**
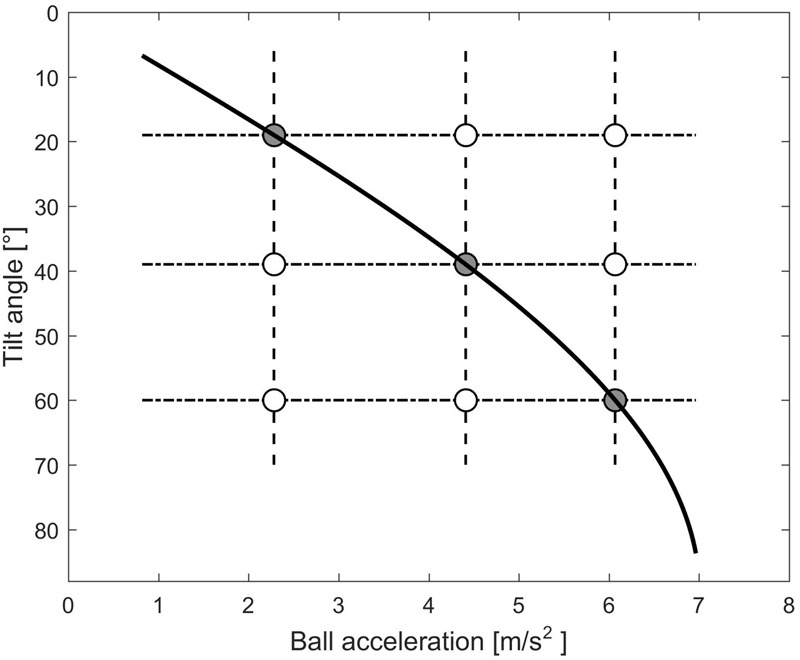
Experimental design for Experiment 1 and 2.

The experimental design is schematically shown in **Figure 2**, where each circle denotes a given combination of ball acceleration *a* and starting tilt angle *𝜃_i,s_*. In this experiment, the observers could manipulate the plane tilt but not the ball acceleration; in other words, starting from any given circle, they could change the displayed conditions only by shifting along the vertical dashed lines in **Figure 2**. Gray circles denote the conditions consistent with physics, that is, when the tilt angle *𝜃_i_* was equal to the target angle *𝜃_b_* congruent with ball acceleration *a*. Therefore, *𝜃_b_* should be chosen by observers whose perceptual judgments were consistent with the dynamics prescribed by Equations 1–2.

**FIGURE 3:**
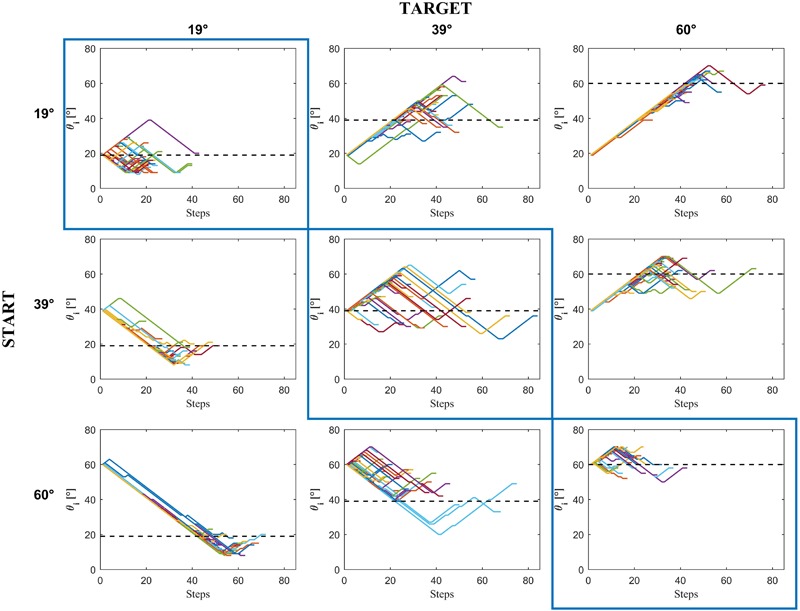
Experiment 1: Incline tilt progression from the starting incline tilt *𝜃_i,s_* to the chosen incline tilt in a representative subject (D.P.) as a function of the number of steps in each condition. Colored traces in each panel are the 15 repetitions × 3 motion durations of each condition.

There were 9 possible starting positions *s_i_* of the ball, 3 for each tilt *𝜃_i,s_*, resulting in 3 different durations of ball motion from the starting position to the lower end of the plane (**Table 1**). In sum, in each experiment there were 9 different combinations of ball accelerations *a* and starting tilt of the plane (3 *𝜃_b_* × 3 *𝜃_i,s_*), 3 motion durations MD (3 *s_i_*), and 15 repetitions for each condition, resulting in a total of 405 trials. All experimental parameters, *𝜃_i,s_, s_i_*, and *𝜃_b_* were randomized across trials in such a manner that there were 1/3 of trials in which *𝜃_i,s_* = *𝜃_b_*, and 2/3 of trials with *𝜃_i,s_*< *𝜃_b_* or *𝜃_i,s_* > *𝜃_b_*, avoiding consecutive trials with the same conditions. Because of the randomization procedure of all 27 different conditions, participants could not memorize specific patterns of stimuli. **Figure 3** shows the sequence of adjustments of the plane tilt made by a representative participant in all repetitions of each condition.

**Table 1:** Parameters of ball motion along the incline for Experiment 1.

Incline			
Angle	Motion duration	Distance (s axis)	Average speed (s axis)
^[∘]^	[ms]	[m]	[m/s]
19	500	0.285	0.570
19	600	0.411	0.684
19	700	0.559	0.798
39	500	0.551	1.102
39	600	0.794	1.323
39	700	1.080	1.543
60	500	0.759	1.517
60	600	1.092	1.821
60	700	1.487	2.124

#### Theoretical Optic Variables

**FIGURE 4:**
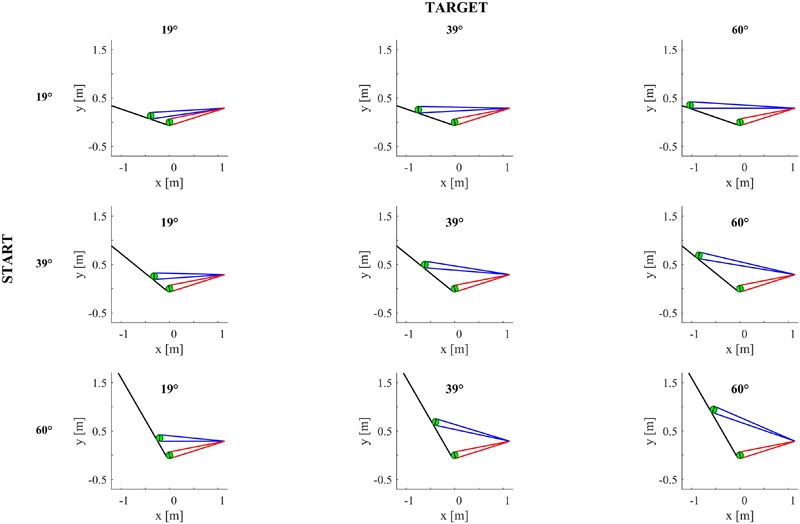
Sagittal view of the inclined plane (long black line) and ball (in green) in all experimental conditions for ball motion duration equal to 0.6 s. The ball is represented twice on the incline, at start position and at incline end. The black ball diameter is orthogonal to the line between ball center and the midpoint between the eyes. Blue and red lines delimit the angular diameter of the ball in the start position and at incline end, respectively, when the viewpoint is the midpoint between participant’s eyes.

**FIGURE 5:**
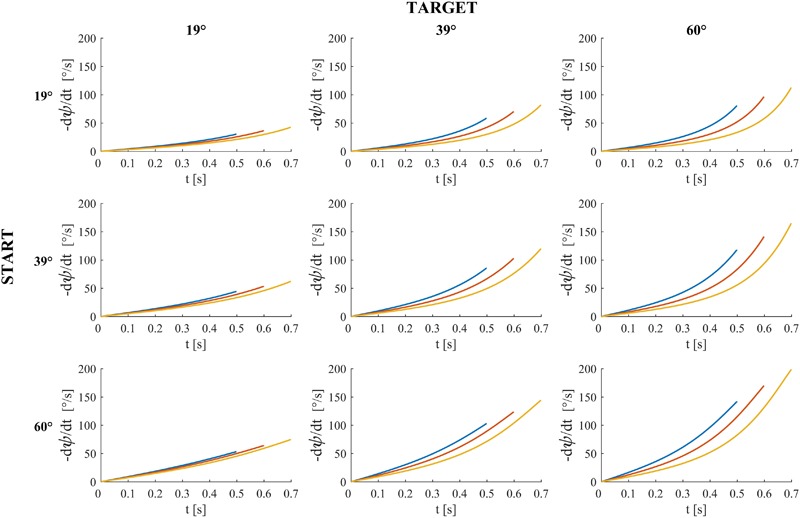
Time derivative of the angular gap *ψ* between the instantaneous position of ball center, the midpoint between the eyes and ball center at incline end for all experimental conditions and motion durations. Blue, red and yellow traces denote *ψ* derivatives (multiplied by –1) for motion duration equal to 0.5, 0.6, and 0.7 s respectively.

**FIGURE 6:**
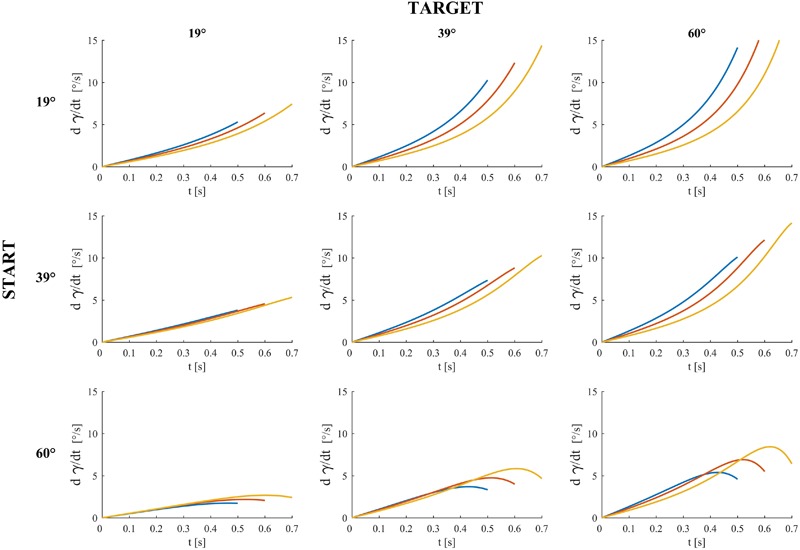
Dilation speed (rate of ball image expansion) for all experimental conditions and motion durations (blue, red, and yellow traces denote dilation speed for motion duration equal to 0.5, 0.6, and 0.7 s respectively). *γ* is the angle the rolling ball subtends at the midpoints between participant’s eyes (i.e., the angular diameter of the ball when the viewpoint is the midpoint between participant’s eyes).

**Figures 4–6** provide estimates of the changes in some optic variables during the descent of the ball. To this end, we assume that the viewpoint of the observer is at the midpoint between the eyes (cyclopean vision). A schematic sagittal view of the ball at the start and end position on the inclined plane is shown in **Figure 4**, for all experimental conditions with MD = 0.6 s. Notice that, for each target tilt angle *𝜃_b_*, the travel distance of the ball on the incline must remain the same irrespective of the starting tilt angle *𝜃_i,s_* to keep ball kinematics constant (compare the plots in each column in **Figure 4**). Instead, for each starting tilt, the travel distance increases with increasing target tilts to keep motion duration constant (compare the plots in each row). **Figure 5** shows the time derivative of the angular gap *ψ* between the instantaneous position of the ball center and its final position on the incline relative to the viewpoint, for all experimental conditions. The absolute value of d*ψ*/dt increases monotonically throughout the descent, faster for shorter motion durations (color-coded, see Figure legend) and greater plane tilts. **Figure 6** shows the time derivative of the angle *γ* subtended by the ball at the viewpoint. It would correspond to the rate of expansion of the cyclopean retinal image (image dilation rate) if the eyes tracked ball motion. Notice that, due to the geometry of the setup, d*γ*/dt increases monotonically throughout the descent, faster for shorter motion durations, with increasing target tilts only for starting angle equal to 19° and 39°, whereas the changes of d*γ*/dt are non-monotonic for starting angle equal to 60°. Indeed, when *𝜃_i,s_* = 60°, d*γ*/dt increases initially and then slightly decreases in the last part of rolling motion, the duration of this phase of decrement being equal to 26–93 ms (depending on MD), 67-94 ms and 70–80 ms for *𝜃_b_* = 19°, 39°, and 60° respectively.

#### Data Analysis and Modeling

Out of a total of 6075 trials (405 trials × 15 subjects), 23 trials were excluded (0.85%) from the analysis due to the presence of artifacts or lack of participant’s attention (as marked in the experiments notebook). In the following, we use a vector notation (boldface) to denote the participants’ responses for the 3 target angles *𝜃_b_* and the 3 starting angles *𝜃_i,s_*. Let the vector **P** indicate the probability **P_***k***,𝜃_***b***_,𝜃_***i,s***__** that participant *k* judged as most natural a ball rolling down with acceleration a (corresponding to target angle *𝜃_b_*) over a plane tilted by *𝜃_i_* degrees relative to the horizontal, when the starting tilt angle was *𝜃_i,s_* (after pooling across repetitions and motion durations). The vector **W** is the associated cumulative distribution function (CDF) **W_***k***,𝜃_***b***_,𝜃_***i,s***__**. Moreover, in order to treat the tilt angles as ordinal random variables, we define the vector **I**= [6, 7, …70] indexing the corresponding values of *𝜃_i_*.

Participants’ responses were analyzed at both population and individual levels. At the population level (responses pooled over all participants and repetitions), the effects of target angle *𝜃_b_* and starting tilt angle *𝜃_i,s_* were estimated in several different ways. First, by computing the median (M) and the interquartile range (IQR) of the index of tilt angle (vector **I**) for which the motion with target angle *𝜃_b_* was judged as the most natural for a given starting tilt angle *𝜃_i,s_*. Second, global estimates of the central tendency index and of the variability were derived from a cumulative probit analysis of **W** (similar to cumulative logit analysis for ordinal responses reported in Agresti, 2007, chapter 6.2). For this second analysis, in the case of *𝜃_b_* equal to 39° or 60°, we fitted a linear function of **I**:

(3) $$\Phi^{-1}[W]\approx \;\beta_{0}+\beta_{1}I$$

Φ^-1^ is the probit link function, β_0_ the intercept and β_1_ the slope of the linear regression. Goodness of fit was assessed by testing that the deviance was not significantly different from 0. In the case of *𝜃_b_* = 19°, instead, the responses were fitted as a function of log(**I**), because they were asymmetrically distributed about the median (see Results):

(4) $$\Phi^{-1}[W]\approx \;\beta_{0}+\beta_{1}\text{log}(I)$$

At individual level, we repeated the cumulative probit analysis of the CDF separately for each participant.

As a further analysis, we applied the Generalized Linear Mixed Model (GLMM) to the data (Moscatelli et al., 2012). The GLMM extends the probit analysis (Equations 3 and 4) to clustered categorical data by separating the overall variability into a fixed and a random component. The fixed component estimates the experimental effects, while the random component estimates the heterogeneity between subjects. For participant *k*, the model was:

(5) $$\Phi^{-}{}^{1}[W_{k}]\approx \;\beta_{0}\;+\;u_{k0}\;+\;(\beta_{1}\;+\;u_{k1})X$$

where **X** is the design matrix including tilt angles (the vector **I**), starting tilt angle (*𝜃_i,s_*), motion duration (MD), and their interactions, β_0_, β_1_ are the fixed-effects coefficients (independent of participants), and u_k0_, u_j1_ are the random-effects coefficients for participant *k*. The dependence on **I** was linear for *𝜃_i,s_* = 39° or 60°, while it was logarithmic for *𝜃_i,s_* = 19°.

Next, global estimates of the central tendency index and of the variability were derived from a probit analysis of **W**. The model was fitted to the CDFs using the R package ‘lme4’ (R 3.2.3, R Development Core Team, 2011)^2^. The significance of the coefficients (two-sided *P*-values) was assessed by means of the Wald statistics:

(6) $$z\text{=}\frac{\overset{\wedge }{\gamma }}{SE}$$

where 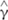 and SE are the parameter estimate and its standard error, respectively. Statistical significance for all tests was set at α = 0.05.

From the CDF, we derived the point of subjective equivalence (PSE) as an estimate of the accuracy of the judgment relative to default physics, and the just-noticeable difference (JND) as an estimate of the precision of the judgment.

#### Statistics

For the condition with target *𝜃_b_* = 19°, the responses (tilt angles *𝜃_i_*) provided by the participants were not normally distributed (Lilliefors test, *P* < 0.001). Therefore, for conformity of analysis across conditions, we report the median M and interquartile range IQR of the responses. Accordingly, the dependence of the responses on motion duration MD was assessed using Kruskal–Wallis non-parametric test separately for each target angle *𝜃_b_* and starting tilt angle *𝜃_i,s_*. Whenever a parameter did not depend significantly on MD, statistical analyses on its M and IQR were performed using Kruskal–Wallis and Ansari–Bradley tests with *𝜃_i_* or *𝜃_i,s_* as a factor (with Bonferroni correction for multiple comparisons). Differences between the response median and the target angle *𝜃_b_* (for each angle *𝜃_b_* and *𝜃_i,s_*) were assessed using Wilcoxon signed ranks or *t*-statistics (*P* < 0.05, level).

Confidence intervals for a median were computed by means of Gaussian based asymptotic approximation of the standard deviation of the median (Kendall and Stuart, 1967).

The degree of skewness of the response distributions was estimated by means of the Pearson’s moment coefficient of skewness: the larger the value, the greater the degree of asymmetry of the distribution.

Data preprocessing was performed with custom software in Matlab (R2015b, Mathworks, Natick, MA, United States), statistical analyses were performed with Matlab and R software (R 3.2.3, R Development Core Team, R foundation for Statistical Computing, Vienna, Austria)^2^.

### Results and Discussion

#### General Statistics

**FIGURE 7:**
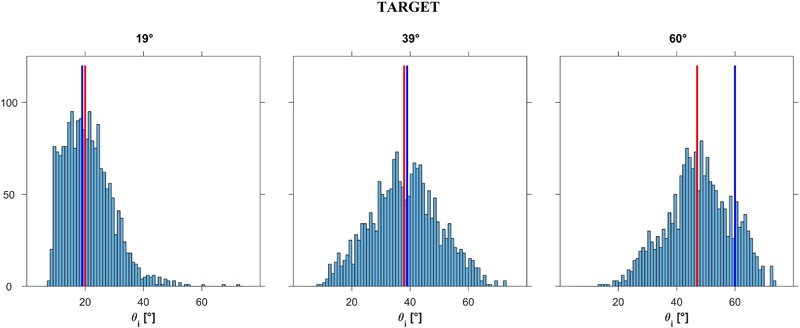
Experiment 1: Distribution histograms of the responses provided after exploration (pooled over participants) for each ball acceleration a*_b_* (i.e., slope *𝜃_b_*). Abscissae: incline tilt *𝜃_i_* for which motion appeared as the most natural for a given a*_b_* (or slope *𝜃_b_*). Ordinates: number of responses. Red bars: distribution medians; blue bars: ideal correct response. In *left, middle, right* panels the data are plotted for slope *𝜃_b_* (TARGET incline) equal to 19°, 39°, or 60°, respectively.

In this experiment, participants were allowed to choose the first presented stimulus as the most natural. In a first analysis, we excluded these *immediate responses* (*n* = 561, about 9% of all trials). **Figure 7** shows the distribution histograms of the responses (*𝜃_i_*) provided by all participants in all trials (*n* = 5491) that involved active exploration of different plane tilts. The responses were pooled together across starting tilt angles *𝜃_i,s_*, motion durations MD and repetitions, for each of the 3 target angles *𝜃_b_* (corresponding to 3 different values of ball acceleration a). Despite the scatter of the responses, systematic trends are evident in the Figure. Thus, the responses depended significantly on target angle *𝜃_b_* (*P* < 0.001): the greater the angle *𝜃_b_*, the greater the tilt angle for which the displayed ball motion was perceived as the most natural. Median values (IQR) of the responses were 20° (12°), 38° (16°), and 47° (15°)^3^ for *𝜃_b_* = 19°, 39°, and 60°, respectively (*n* = 1871, 1819, and 1801, respectively). These median values were very close to the target values for *𝜃_b_* = 19° and 39°. Instead, the median values were considerably smaller than the target value for *𝜃_b_* = 60°, indicating that the participants tended to associate the highest tested acceleration of the ball with a slope less steep than that consistent with physics. The scatter diagrams of **Figure 7** also show that, while the responses were distributed roughly symmetrically about the median for *𝜃_b_* = 39° and 60° (skewness coefficient equal to 0.048 and -0.156 respectively), in the case of *𝜃_b_* = 19° the response distribution was highly asymmetrical (skewness coefficient = 0.967).

Next, we analyzed all responses together (*n* = 6052), including the *immediate responses*. At the population level, we found a significant difference (*P* < 0.001) between the responses for the 3 target angles *𝜃_b_*, as well as a significant attractive effect (*P* < 0.001) of the starting tilt angle *𝜃_i,s_* on the median of the responses. Instead, neither motion duration nor repetition had major effects. In fact, we found that motion duration significantly affected only a subset of the responses for *𝜃_b_* = 60° and *𝜃_i,s_* = 19° (*P* = 0.039) or 39° (*P* = 0.007); specifically, the median response for MD = 0.5 and 0.7 s were significantly different for *𝜃_i,s_* = 19° and *𝜃_i,s_* = 39°, while the median response for MD = 0.5 and 0.6 s were significantly different for *𝜃_i,s_* = 39°. However, the size of these effects was quite small, the difference between maximum and minimum median values being equal to 3°. At the population level, there was no significant effect of repetition on the responses (Kruskal–Wallis, *P* = 0.12).

**Table 2** reports, separately for each target angle *𝜃_b_* and starting angle *𝜃_i,s_*, the median values, IQR and 95% confidence intervals of the responses pooled across motion durations, repetitions and participants. It can be seen that the median value of the responses shifted to higher values of *𝜃_i_* with increasing values of *𝜃_i,s_*, for all target angles *𝜃_b_*. However, this effect was more limited for *𝜃_b_* = 19°, whose 3 median responses were very close to the target angle (within about 3°, corresponding to 3 adjustment steps of the procedure, see Methods). The deviation from the target angle was slightly greater for *𝜃_b_* = 39° (median values within about 6° of the target), and even more substantial for *𝜃_b_* = 60° (median values within about 18° of the target). Notice that, for all target angles *𝜃_b_*, including 60°, the median values for the condition *𝜃_i,s_*, = *𝜃_b_* was fairly close to the target value itself.

**Table 2:** Experiment 1: Median over all repetitions of the average subject responses for *𝜃_b_* = 19°, 39°, and 60° and *𝜃_i,s_* = 19°, 39°, and 60°.

*𝜃_b_*	*𝜃_i,s_*	Median	IQR	Inferior CI	Superior CI
19°	19°	18°20′	2°04′	17°51′	18°49′
	39°	21°08′	2°16′	20°36′	21°40′
	60°	22°12′	2°40′	21°35′	22°49′
39°	19°	33°32′	3°20′	32°45′	34°19′
	39°	38°20′	2°50′	37°40′	38°60′
	60°	43°12′	5°00′	42°02′	44°22′
60°	19°	42°12′	3°53′	41°17′	43°07′
	39°	46°40′	2°18′	46°08′	47°12′
	60°	54°16′	3°09′	53°32′	55°00′

*IQR: interquartile range. CI: 95% confidence intervals.*

#### Probit Analysis

The results were confirmed by a cumulative probit analysis of the population CDFs of the responses. Thus, we found a significant difference (Kolmogorov–Smirnov, *P* < 0.001) between the CDFs for the three target *𝜃_b_* values. Estimated medians (JND) were 18.72° (5.76°), 37.96° (8.31°), and 46.83° (7.65°) for *𝜃_b_* = 19°, 39°, and 60°, respectively. For each *𝜃_b_*, the CDFs had the same shape (two-sample Kolmogorov–Smirnov for each pairing, *P* > 0.31), the same median value (Kruskal–Wallis, *P* > 0.90), and the same variance (Ansari–Bradley, *P* > 0.11) for all motion durations. For any given target angle, except for *𝜃_b_* = 19° with *𝜃_i,s_* = 39° and 60°, there was a significant difference (Kolmogorov-Smirnov, *P* < 0.001) between the CDFs of the responses for the 3 starting angles *𝜃_i,s_*. For any given starting angle, there was a significant difference (Kolmogorov-Smirnov, *P* < 0.001) between the CDFs of the responses for the three target angles.

#### GLMM Model

The effects of the experimental factors were investigated further by modeling the individual CDFs of the responses using the GLMM (Equation 5). First, we searched for any significant effect of motion duration on the median and slope for each value of *𝜃_b_* and *𝜃_i,s_*, and we found none (Wald Statistics, *P* > 0.14). Accordingly, we further used a GLMM model with only 2 fixed effects (*𝜃_b_* and *𝜃_i,s_*), their interactions, and random effects for the intercept, *𝜃_b_* and *𝜃_i,s_*.

**FIGURE 8:**
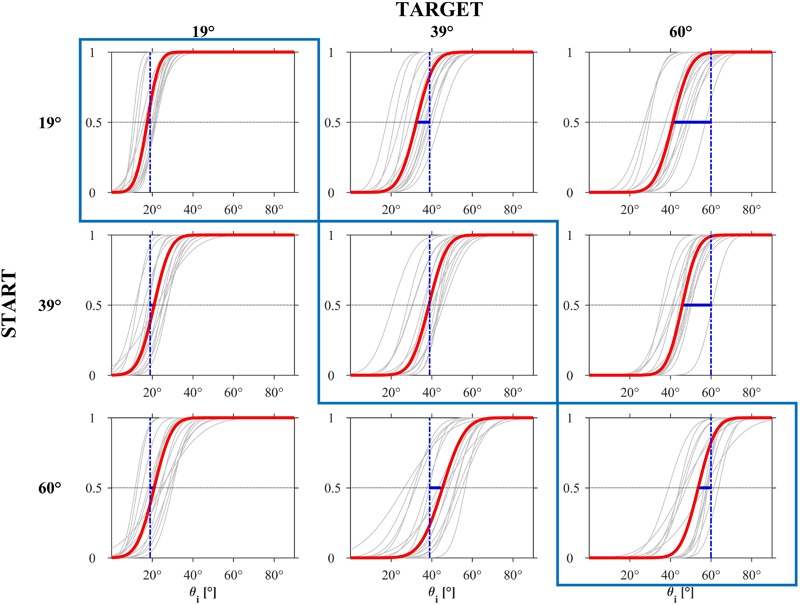
Experiment 1: CDFs estimated by the GLMM for each participant (gray) and for the population (red). The blue bar represents the distance between the PSE and the ideal correct response.

**Figure 8** (based on all trials including the *immediate responses*) shows the estimated CDFs for each participant (black curves) and for the population (red curves). The results in this Figure are plotted with the same format as that of the experimental design in **Figure 2**. Clearly, the responses were more consistent among participants for *𝜃_b_* = 19° than for the other two *𝜃_b_* values.

**Table 3** reports, for each *𝜃_b_*, the estimated PSE and JND of the population CDFs. The *t*-tests performed over all participants showed that the estimates of PSE for *𝜃_b_* = 19° and for all *𝜃_i,s_* values were not significantly different from the target value of 19° (*P* > 0.09), the PSE for *𝜃_b_* = 39° and *𝜃_i,s_* ≥ 39° were not significantly different from the target value of 39° (*P* > 0.11), whereas the PSE for *𝜃_b_* = 60° and for all *𝜃_i,s_* were significantly lower than the target value of 60° (one tailed *t*-test, *P* < 0.002). Also this analysis confirms that for all target angles *𝜃_b_*, including 60°, the PSE values for the condition *𝜃_i,s_*, = *𝜃_b_* was fairly close to the target value itself.

**Table 3:** Experiment 1: Median values (PSE) and JND of the population CDFs estimated by GLMM for *𝜃_b_* = 19°, 39°, and 60° and *𝜃_i,s_* = 19°, 39°, and 60°.

		PSE				JND			
***𝜃_b_***	***𝜃_i,s_***	**Estimate**	***SE***	**Inferior CI**	**Superior CI**	**Estimate**	***SE***	**Inferior CI**	**Superior CI**
			
19°	19°	17°06′	1°08′	14°31′	19°20′	3°07′	0°13′	2°38′	3°30′
	39°	20°11′	1°19′	17°38′	22°39′	4°18′	0°17′	3°46′	4°59′
	60°	20°28′	1°40′	17°24′	24°15′	4°33′	0°24′	3°49′	5°25′
39°	19°	31°56′	1°60′	28°02′	35°51′	4°35′	0°16′	4°05′	5°05′
	39°	38°15′	1°42′	34°56′	41°34′	4°38′	0°19′	4°00′	5°15′
	60°	44°33′	2°30′	39°39′	49°26′	5°58′	0°30′	4°60′	6°56′
60°	19°	41°19′	2°16′	36°54′	45°45′	4°48′	0°17′	4°14′	5°22′
	39°	45°57′	1°34′	42°52′	49°01′	4°12′	0°18′	3°37′	4°47′
	60°	54°06′	1°47′	50°36′	57°35′	4°33′	0°26′	3°42′	5°23′

*SE: standard error. CI: 95% confidence intervals.*

The discrimination precision (JND) was about 4° for all target tilts, corresponding to about 6% over the explorable range of plane tilts (6°/70°). For *𝜃_b_*≤ 39° there was a small but significant effect of *𝜃_i,s_* on the estimated JND of the CDF. For *𝜃_b_* = 19° the estimated JND of the CDF for *𝜃_i,s_* = 19° (≈3°) was significantly smaller than that for the other *𝜃_i,s_* (≈4°, one tailed *t*-test, *P* < 0.003); for *𝜃_b_* = 39° the estimated JND of the CDF for *𝜃*_s_≤ 39° (≈5°) was significantly smaller than that for *𝜃_i,s_* = 60° (≈6°, one tailed *t*-test, *P* < 0.006) whereas for *𝜃_b_* = 60° the JNDs (≈5°) were not significantly different for the 3 *𝜃_i,s_* (*P* > 0.24).

#### Immediate Responses

As noticed earlier, in 561 trials the participants judged the first presented stimulus as the most natural, without any adjustment of the plane tilt and without any re-observation of ball motion. Had these *immediate responses* been provided randomly, their distribution should have been uniform, since the probability of each given starting tilt *𝜃_i,s_* was uniform (equal to 1/3). Instead, we found that the distribution was highly non-uniform: the rate of *immediate responses* for *𝜃_b_* = 19° (*N* = 147, across all participants) was 80.95%, 17.69%, and 1.36% for *𝜃_i,s_* = 19°, 39°, or 60° respectively, for *𝜃_b_* = 39° (*N* = 197) it was 18.27%, 35.03%, and 46.70% respectively, and for *𝜃_b_* = 60° (*N* = 217) it was 7.83%, 29.95%, and 62.21% respectively. Thus, peak rate occurred at the starting tilt equal to the target tilt for *𝜃_b_* = 19° and *𝜃_b_* = 60°, while it occurred at a starting tilt greater than the target tilt for *𝜃_b_* = 39°.

### Conclusion and Discussion

Within the wide range of possible plane tilts, participants often picked a tilt that was roughly consistent with the dynamics of the displayed ball motion, as defined by the hidden variable of target tilt. However, there was a systematic effect of the initial conditions: the match between the chosen plane tilt and the ball acceleration (corresponding to the target tilt) was generally better when the trial started from the combination of tilt and acceleration corresponding to Newtonian mechanics than when it started far away from it. In addition, the physically congruent starting conditions were judged as natural at the beginning of a trial more often than the physically incongruent starting conditions, as shown by the higher percentage of immediate responses provided by the participants without any exploration of the stimuli space. We cannot rule out that some immediate responses were simply due to hasty, little pondered responses, but even so their distribution should have been uniform across starting tilt angles.

Perceptual judgments inconsistent with physics were more frequent for the target tilt of 60° than for the smaller tilts. The differential performance as a function of target tilt cannot be explained easily in terms of the optic cues that potentially contribute to perceptual discrimination of the moving ball. In fact, both the absolute value of the rate of change of the angular gap between the instantaneous and the final position of the ball on the incline (**Figure 5**), as well as the rate of change of the visual angle subtended by the ball (**Figure 6**) were greater for the target tilt of 60° than for the smaller target tilts. In theory, higher values of the optic variables within a physiological range should lead to better discriminations than low values of the optic variables.

Instead, the underestimate of the steepest target slope (60°), coupled with a correct estimate of the shallowest slope (19°), might depend on a linearization of the expected relationship between ball acceleration and tilt angle, since ball acceleration varies with the sine of the tilt angle (see **Figure 2**). Moreover, the target tilt of 60° involved the highest acceleration of the ball of those employed in the protocol. Therefore, to test the role of acceleration directly, we designed a new experiment that was complementary to Experiment 1, in the sense that this time the participants were asked to modify the acceleration of the rolling ball until the resulting motion looked natural for a given slope (acceleration adjustment instead of slope adjustment as in Experiment 1).

## Experiment 2

In each trial, the ball rolled down the plane tilted by a preset angle of 19°, 39°30′ or 60°, with an acceleration that was generally different from that consistent with the tilt of the plane. Participants adjusted iteratively the ball acceleration within a range corresponding to a theoretical tilt of the plane between 6°42′ and 84°36′, until they judged that the ball motion with the newly set acceleration was the most natural of all tested motions for the preset tilt of the plane.

### Methods

#### Participants

Fifteen subjects (nine females and six males, 24.5 ± 8.9 years old, mean ± SD) participated in this experiment. Five of them had previously participated in Experiment 1 about 4 months before.

#### Apparatus, Visual Stimuli, and Procedures

The apparatus, stimuli, and procedures were similar to those of Experiment 1, with the following changes. In each trial, the plane in the 3D scenario was shown with one of 3 tilt angles *𝜃_i:_* 19°, 39°30′^4^, or 60°. In contrast with Experiment 1, here the plane tilt was not modified during the trial. The target was represented by the acceleration that the ball should have had on that specific incline to be consistent with Newtonian mechanics. Ball kinematics obeyed Equations 1–2 as in Experiment 1. At trial start, the acceleration a*_b,s_* of the ball center of mass was consistent with the acceleration at one of 3 tilt angles *𝜃_b,s_* 19°, 39°30′, or 60°. *𝜃_b,s_* was the same or different relative to the tilt of the plane *𝜃_i_* actually shown, depending on the trial. As in Experiment 1, there were 9 possible starting positions *s_i_* of the ball, 3 for each tilt *𝜃_i_*, resulting in 3 different durations (0.5, 0.6, or 0.7 s) of ball motion from the starting position to the lower end of the plane. Participants were instructed to observe the ball motion and then to increase or decrease the acceleration of the ball by pressing the corresponding button of the Wand, until the motion on the incline appeared as the most natural one. The iterative adjustment of acceleration occurred in steps equivalent to a (hidden) tilt of the plane *𝜃_b_* by ±4°6′ relative to the previous inclination, within the range 2°36′/84°36′, starting from the initial value *𝜃_b,s_*. Therefore, in each trial, ball acceleration was:

(7) $$a_{b}=\frac{5}{7}\times g\times \sin(\theta_{b})=\frac{5}{7}\times g\times \sin(\theta_{b,s}+(n_{I}-n_{D})\times \delta )\;\left(7\right)$$

where *_I_ (n_D_)* is the number of times the Increase (Decrease) button was clicked in a trial, and δ = 4°6′ is the step size. At trial start *_I_ = n_D_* = 0. Because of the allowable tilt range, the range for acceleration was 0.032*g* ≤ a*_b_* ≤ 0.711 *g*. Notice that the amount of change of ball acceleration after a given click depended on the actual ball acceleration and the angular step *δ* according to:

(8) $$\Delta a_{b}=\frac{10}{7}\times g\times \cos(\theta_{b}+\frac{\delta }{2})\times \sin(\frac{\delta }{2})$$

Therefore, the smaller the actual acceleration, the greater was the amount of acceleration change.

If the participant tried to go below the allowed range in 3 consecutive attempts, the trial was terminated and the value of *𝜃_b_* = 2°36′ was taken as the response (the upper value of the range was never exceeded). Moreover, the participants could choose the first presented stimulus as the most natural, without any adjustment of ball motion.

The experimental design is schematically shown in **Figure 2**, where each circle denotes a given combination of ball acceleration a*_b,s_* and tilt angle *𝜃_i_*. In this experiment, the observers could manipulate the ball acceleration but not the plane tilt; in other words, starting from any given circle, they could change the displayed conditions only by shifting along the horizontal, dash-dotted lines in **Figure 2**. Gray circles denote the conditions consistent with physics, that is, when the angle *𝜃_b_* corresponding with ball acceleration a*_b,s_* coincided with the tilt angle *𝜃_i_*. Thus, *𝜃_b_* = *𝜃_i_* represents the tilt angle that would be chosen by an observer whose perceptual judgments were consistent with the dynamics prescribed by Equations 1–2.

In each experiment, there were 9 different combinations of starting ball accelerations a*_b,s_* and tilt of the plane (3 *𝜃_b,s_* × 3 *𝜃_i_*), 3 motion durations MD (3 *s_i_*), and 15 repetitions for each condition, resulting in a total of 405 trials. All experimental parameters, *𝜃_i_, s_i_*, and *𝜃_b,s_*, were randomized across trials in such a manner that there were 1/3 of trials in which *𝜃_b,s_* = *𝜃_i_*, and 2/3 of trials with *𝜃_b,s_*< *𝜃_i_* or *𝜃_b,s_* > *𝜃_i_*, avoiding consecutive trials with the same conditions.

**FIGURE 9:**
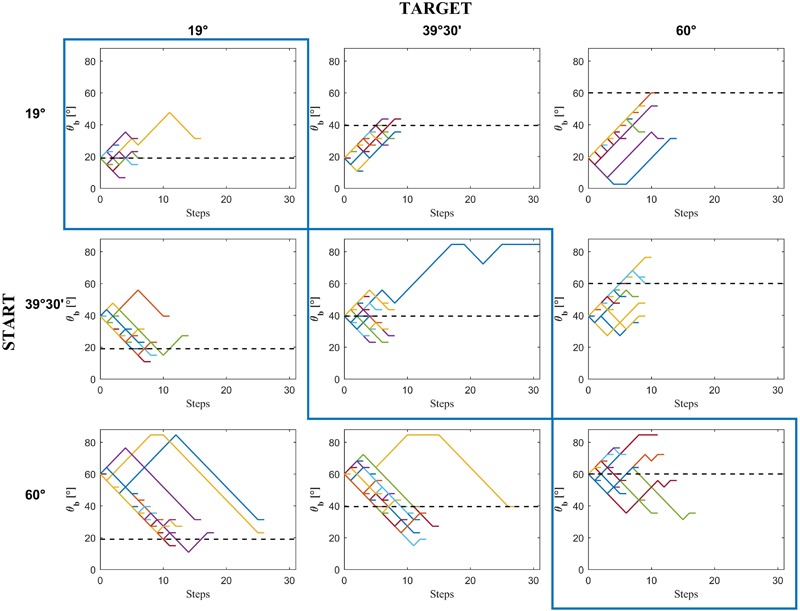
Experiment 2: Progression from the START condition to the chosen motion in a representative subject (C.C.). Colored traces in each panel are the 15 repetitions × 3 motion durations of each condition.

On average, reaching a final decision required 8 adjustments, and the experimental session lasted 1 h and 39 min. **Figure 9** shows the sequence of adjustments of the ball acceleration (expressed in tilt angles) made by a representative participant in all repetitions of each condition.

#### Data Analysis, Modeling and Statistics

Data processing was identical to that of Experiment 1, taking into account that the nature of the dependent and independent variables reflected the specific protocol.

### Results and Discussion

#### General Statistics

**FIGURE 10:**
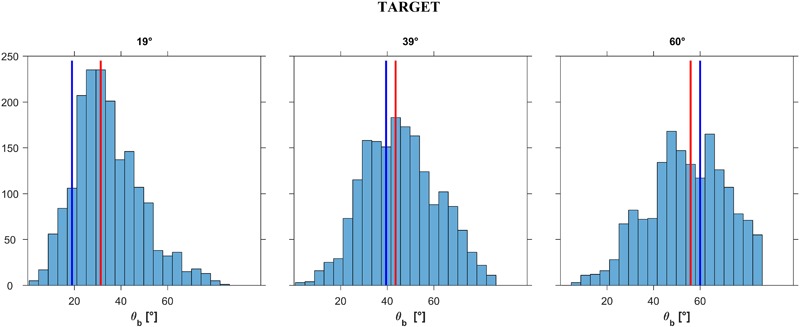
Experiment 2: Distribution histograms of the responses (pooled over participants) for each incline tilt *𝜃*. Abscissae: *𝜃_b_* for which motion appeared as the most natural for the given incline tilt *𝜃_i_* (TARGET incline). Ordinates: number of responses. Red bars: ideal distribution medians; blue bars: correct response.

**Figure 10** shows the distribution histograms of the responses (*𝜃_b_*) provided by all participants in all trials that involved active exploration of different accelerations (*n* = 5227, excluding 848 *immediate responses*). We pooled together the responses across starting accelerations a*_b,s_* or equivalently *𝜃_b,s_*, motion durations MD and repetitions, for each of the 3 target angles *𝜃_i_*. We found that the responses depended significantly on target angle *𝜃_i_* (Kruskal–Wallis, *P* < 0.001): the greater the angle *𝜃_i_*, the greater the acceleration and corresponding tilt angle for which the displayed ball motion was perceived as the most natural. Median values (IQR) of the responses were 31°18′ (20°30′), 43°36′ (20°30′) and 55°54′ (24°36′)^5^ for *𝜃_i_* = 19°, 39°30′ and 60°, respectively (*n* = 1784, 1779 and 1664, respectively). These median values were reasonably close to the target values for *𝜃_b_* = 39°30′ and 60°. Instead, the median values were considerably larger than the target value for *𝜃_b_* = 19°, indicating that the participants tended to associate the lowest tested acceleration of the ball with a slope steeper than that consistent with physics.

Next, we analyzed all responses together (*n* = 6075), including the *immediate responses*. At the population level, we found a significant difference (Kruskal–Wallis, *P* < 0.001) between the responses for the 3 target angles *𝜃_i_*, as well as a significant attractive effect (*P* < 0.001) of the starting acceleration a*_b,s_* or equivalently *𝜃_b,s_* on the median of the responses. There was also a significant (*P* < 0.01) effect of motion duration at the population level, but this was small and driven by only two participants. At individual level, MD had a significant (*P* < 0.01) effect on the responses (computed separately for each starting *𝜃_b,s_* and incline tilt *𝜃_i_*) of only two subjects. At the population level, there was no significant effect of repetition on the response median of each condition (Kruskal–Wallis, *P* > 0.05).

**Table 4** reports the median values, IQR and 95% confidence intervals of the responses pooled across motion durations, repetitions and participants. As in Experiment 1, the median value of the responses shifted to higher values with increasing starting values of tilt, for all target angles. These results were confirmed by a cumulative probit analysis of the population CDFs of the responses. For any given target angle *𝜃_i_*, there was a significant difference (Kolmogorov–Smirnov, *P* < 0.001) between the CDFs for the 3 starting *𝜃_b,s_*. For any given starting *𝜃_b,s_*, there was a significant difference (Kolmogorov–Smirnov, *P* < 0.001) between the CDFs of the responses for the 3 incline tilts. For any given target incline *𝜃_i_*, the estimated medians of the responses tended to increase monotonically with the starting *𝜃_b,s_*.

**Table 4:** Experiment 2: Median over all repetitions of the average subject responses for *𝜃_i_* = 19°, 39°30′, and 60° and *𝜃_b,s_* = 19°, 39°30′, and 60°.

*𝜃_i_*	*𝜃_b,s_*	Median	IQR	Inferior CI	Superior CI
19°	19°	25°50′	3°37′	24°59′	26°41′
	39°30′	33°39′	4°00′	32°42′	34°35′
	60°	41°51′	4°46′	40°44′	42°58′
39°30′	19°	35°59′	6°08′	34°33′	37°25′
	39°30′	46°20′	6°35′	44°47′	47°53′
	60°	55°09′	6°35′	53°37′	56°42′
60°	19°	45°39′	6°48′	44°03′	47°15′
	39°30′	54°44′	5°08′	53°32′	55°56′
	60°	64°22′	3°06′	63°39′	65°06′

*IQR: interquartile range. CI: 95% confidence intervals.*

#### GLMM Model

**FIGURE 11:**
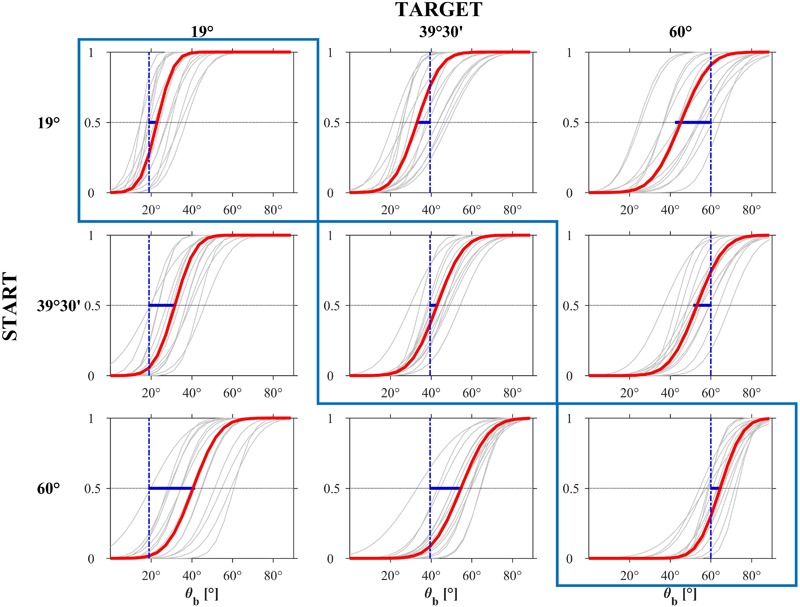
Experiment 2: CDFs estimated by the GLMM for each participant (gray) and for the population (red). The blue bar represents the distance between the PSE and the ideal correct response.

The effects of the experimental factors were investigated further by modeling the individual CDFs of the responses using the GLMM. Since there was no significant effect (Wald Statistics, *P* > 0.56) of motion duration on the median and slope for each value of *𝜃_i_* and starting *𝜃_b,s_*, we used a GLMM model with 2 fixed effects (starting *𝜃_b,s_* and target *𝜃_i_*), their interactions, and random effects for the intercept, *𝜃_b,s_* and *𝜃_i_*. **Figure 11** shows the estimated CDFs for each participant (black curves) and for the population (red curves) separately for each condition (based on all trials including the *immediate responses*). The responses tended to be more consistent across participants and the estimated PSE of the population CDFs was closer to the target value when target incline *𝜃_i_* was congruent with *𝜃_b,s_* than when it was incongruent. **Table 5** reports, for each *𝜃_i_* and *𝜃_b,s_*, the estimated PSE and JND of the population CDFs. The *t*-tests performed over all participants showed that the estimates of PSE for *𝜃_i_* = *𝜃_b,s_* were not significantly different from the target incline *𝜃_i_* (*P* > 0.01), as was the estimate of PSE for *𝜃_i_* = 39°30′ and *𝜃_b,s_* = 19° (*P* > 0.05).

**Table 5:** Experiment 2: Median values (PSE) and JND of the population CDFs estimated by GLMM for *𝜃_i_* = 19°, 39°30′, and 60° and *𝜃_b,s_* = 19°, 39°30′ and 60°.

		PSE				JND			
*𝜃_i_*	*𝜃_b,s_*	Estimate	*SE*	Inferior CI	Superior CI	Estimate	*SE*	Inferior CI	Superior CI
19°	19°	22°45′	1°49′	19°11′	26°20′	4°23′	0°23′	3°37′	5°09′
	39°30′	31°35′	2°05′	27°31′	35°40′	5°20′	0°21′	4°39′	6°01′
	60°	40°22′	3°06′	34°17′	46°26′	6°55′	0°20′	6°17′	7°34′
39°30′	19°	33°22′	2°13′	29°02′	37°41′	6°14′	0°38′	4°59′	7°28′
	39°30′	43°16′	1°46′	39°49′	46°43′	6°54′	0°24′	6°08′	7°41′
	60°	54°54′	2°04′	50°52′	58°57′	7°32′	0°30′	6°34′	8°30′
60°	19°	44°03′	3°22′	37°27′	50°40′	7°38′	0°30′	6°40′	8°36′
	39°30′	52°36′	2°10′	48°22′	56°50′	7°08′	0°29′	6°11′	8°06′
	60°	63°52′	1°33′	60°50′	66°55′	6°04′	0°28′	5°10′	6°59′

*SE: standard error. CI: 95% confidence intervals.*

The discrimination precision (JND) ranged between about 4° and 7°, that is, 5% to 9% of the range of plane tilts (about 3°/84°) corresponding to the explorable accelerations. The JNDs of *𝜃_i_* = 19° and 60° for the corresponding *𝜃_b,s_* were significantly (*P* < 0.05) smaller than for the other values of *𝜃_b,s_* (one tailed *t*-test, *P* < 0.01 and *P* < 0.001 respectively). The JND of *𝜃_i_* = 39°30′ for the corresponding *𝜃_b,s_* was not significantly different from that for the other values of *𝜃_b,s_* (*t*-test, *P* > 0.2). Notice that, for *𝜃_i_* = 19° and *𝜃_i_* = 39°30′, the JNDs increased monotonically for increasing values of *𝜃_b,s_* while the opposite was true for *𝜃_i_* = 60°.

#### Immediate Responses

As in Experiment 1, the *immediate response* rate for *𝜃_i_* = 19° and *𝜃_i_* = 60° was higher when *𝜃_b,s_* was consistent with target tilt *𝜃_i_*. Instead, for *𝜃_i_* = 39°30′ the rate was higher for *𝜃_b,s_* inconsistent with target tilt.

Specifically, the rate of *immediate responses* for *𝜃_b_* = 19° (*N* = 223, across all participants) was 43.50%, 28.70%, and 27.80%, for *𝜃_i,s_* = 19°, 39°30′ or 60° respectively, for *𝜃_b_* = 39°30′ (*N* = 217) it was 17.51%, 35.48%, and 47.00%, respectively, and for *𝜃_b_* = 60° (*N* = 201) it was 11.44%, 35.82%, and 52.74%, respectively.

### Conclusion and Discussion

In general, participants tended to choose a value of ball acceleration that was roughly consistent with the inclination of the displayed plane. As in the previous experiment, the match between the chosen ball acceleration and the plane tilt was often better when the trial started from the right combination of acceleration and tilt than when it started far away from it. Moreover, the physically congruent starting conditions were judged as natural at the beginning of a trial more often than the physically incongruent starting conditions, as shown by the distribution of immediate responses. In contrast with Experiment 1, however, judgments inconsistent with physics were more frequent for the lowest tested acceleration of the ball, corresponding to a target tilt of 19°, instead of the highest tested acceleration as in Experiment 1. In line of principle, this result could depend on the lower values of the optic variables that potentially contribute to perceptual discrimination of the moving ball, since both the absolute value of d*ψ*/dt (**Figure 5**) and the value of d*γ*/dt (**Figure 6**) were smaller for the tilt of 19° than for the greater tilts.

## Experiment 3

In both previous experiments, we found a significant effect of the initial conditions: accuracy was higher when the participants started the exploration from the combination of tilt and acceleration corresponding to the conditions consistent with physics than when they started far away from those conditions. As a control, we carried out a third experiment similar to Experiment 1 (slope adjustment), but avoiding the coincidence of starting condition and target. In other words, the acceleration of the ball was always inconsistent with the initial plane tilt. Moreover, here we used a two-alternative forced-choice (2-AFC) paradigm with an adaptive staircase for the adjustment of the incline angle, by asking the observers to indicate if the tilt of the incline was higher or lower than the tilt consistent with the ball kinematics displayed in that trial. This paradigm avoided asking to choose one specific combination of tilt and acceleration as the most natural.

### Methods

#### Participants

Fifteen subjects (10 females and 5 males, 39.3 ± 7.1 years old, mean ± SD) participated in this experiment. Four of them had previously participated in Experiment 1 (about 1 year before).

#### Apparatus, Visual Stimuli, and Procedures

The apparatus, stimuli and procedures were similar to those of Experiment 1, with the following changes. Instead of the Wand, participants held a cylindrical plastic object in the right hand (12 cm × 3 cm, length × diameter; 60 g weight), with a pin (hitter) protruding between the index and middle finger. The position and orientation of the cylinder in 3D was monitored by means of the Vicon system at 100 Hz (10 Bonita cameras), so that a virtual cylinder with a hitter were displayed in the 3D scene in the same position and orientation as the real object. A trial started once the tip of the hitter reached a virtual sphere (9 cm diameter) placed 25 cm below, 10 cm to the right, and 1 m in front of the lower end of the incline. Before each trial start, the plane in the 3D scene was shown tilted by 40° (i.e., *𝜃_i,s_* = 40°). After a pseudorandom delay of 500 or 700 ms from trial start, the ball rolled down the plane from initial position *s_i_*, with the law of motion described by Equations 1 and 2 and with an acceleration a such that the corresponding target tilt angle *𝜃_b_* was either 25° or 55°, depending on the trial. Therefore, in this experiment, the acceleration a of the ball (and the corresponding target angle *𝜃_b_*) was always inconsistent with the initial plane tilt *𝜃_i,s_*. Moreover, both target tilts *𝜃_b_* were spaced apart from the starting tilt *𝜃_i,s_* by the same angle (±15°). There were 4 different starting positions *s_i_*, and 15 repetitions for each condition for a total of 60 trials. Two different values of *s_i_* were associated with each acceleration, resulting in 2 ball motion durations (0.5 or 0.7 s) from the starting position to the lower end of the incline (see **Table 6**). *𝜃_b_* and *s_i_* were randomized across trials, avoiding consecutive trials with the same conditions.

**Table 6:** Parameters of ball motion along the incline for Experiment 3.

Incline			
**Angle**	**MD**	**Distance (s axis)**	**Average speed (s axis)**

^[∘]^	[ms]	[m]	[m/s]
25	500	0.370	0.740
25	700	0.726	1.037
55	500	0.718	1.435
55	700	1.406	2.009

Participants were asked to decide if the tilt of the currently displayed plane was greater or smaller than the natural tilt for the observed motion (2-AFC task). To this end, at ball disappearance, two response boxes with different sizes were displayed in the 3D scene. By moving the hand with the hitter inside the greater (smaller) box, the participant indicated that the plane tilt was greater (smaller) than the natural tilt for the observed motion. The greater box (13.5 cm side) and the smaller box (10.13 cm side) were placed 10 cm to the left and right of the plane, respectively. Both boxes were placed 10 cm below and 70 cm in front of the lower end of the incline. Once selected, the box changed color (from white to red) to signal the acquisition of the participant’s response. No feedback about response accuracy was provided. After the participant’s choice, the tilt angle *𝜃_i_* of the plane was increased (when the response was “smaller”) or decreased (when the response was “greater”) by a given step size according to a PEST staircase (Taylor and Creelman, 1967). The initial step size was 8°. Afterward, the step size was doubled after two consecutive responses in the same direction (both “greater” or both “smaller”), while it was halved after each inversion (from “greater” to “smaller” or viceversa). The maximum step size was 16°, and the allowed range for *𝜃_i_* was 8°–72°, Whenever the participant’s responses would have brought the tilt angle outside this range, the plane tilt was reset to *𝜃_i,s_* = 40°. Each trial ended when the step size first reduced to 0.25°. The last value of tilt angle at the end represented the participant’s response and was stored together with the preceding sequence of changes.

Before the experiment, participants received written instructions. Afterward, the experimenter performed 5 trials to demonstrate the setup. In these trials, the stimuli were unrelated to the experimental ones (different initial plane tilts and accelerations), and the experimenter provided random responses not to provide any information about the criterion for choosing one or another plane tilt.

#### Data Analysis and Statistics

All 900 trials (60 trials × 15 subjects) were included in the analysis. The responses (last values of tilt angles *𝜃_i_*) were analyzed at both population level (responses pooled over all participants and repetitions) and individual level (responses pooled over all repetitions). As before, we report median M and interquartile range IQR of all responses. Accordingly, the dependence of the responses on motion duration MD was assessed using Kruskal–Wallis non-parametric test separately for each target angle *𝜃_b_*. Whenever the responses did not depend significantly on MD, statistical analyses on M and IQR were performed using Kruskal–Wallis and Ansari–Bradley tests with *𝜃_i_* as a factor (with Bonferroni correction for multiple comparisons). Differences between the response median and the target angle *𝜃_b_* (for each angle *𝜃_b_*) were assessed using Wilcoxon signed ranks or t-statistics (*P* < 0.05, level).

### Results and Discussion

**FIGURE 12:**
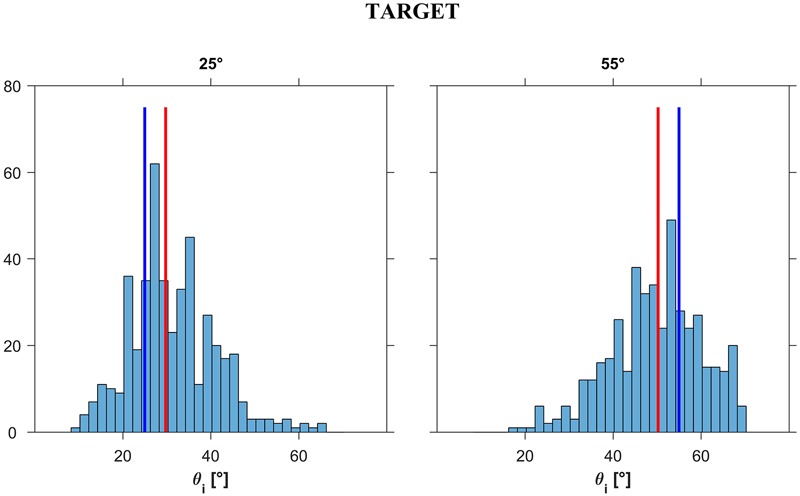
Experiment 3: Distribution histograms of the responses (pooled over participants) for each ball acceleration a_b_ (i.e., slope *𝜃*_b_). Abscissae: incline tilt for which motion appeared as correct for a given a_b_ (i.e., slope *𝜃*_b_). Ordinates: number of responses. Red bars: distribution medians; blue bars: ideal correct response.

**FIGURE 13:**
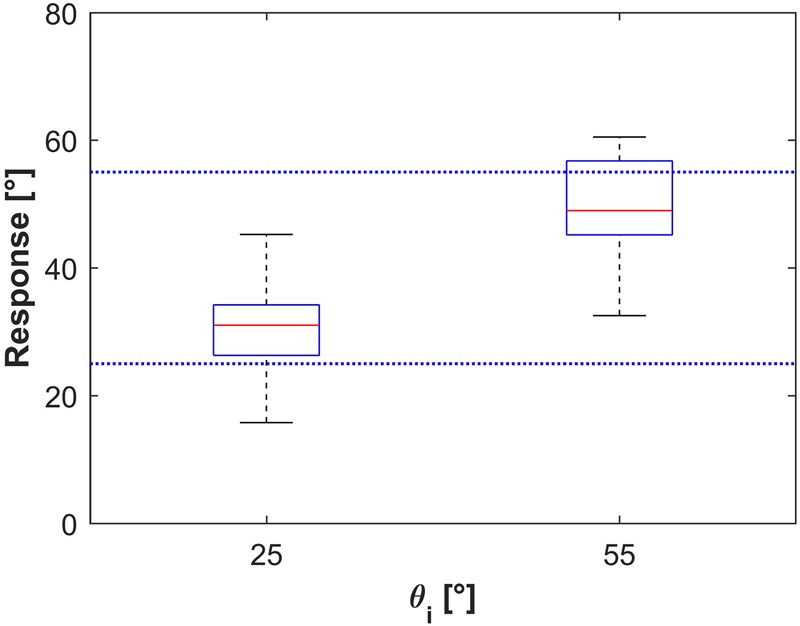
Experiment 3: Box-and-whisker plots of all subject responses. Bottom and top of the boxes correspond to the lower and upper quartile, respectively, and define the IQR. The red line in each box is the median between subjects’ median response. Results from all repetitions of any subjects have been pooled for each condition. The lower and upper ends of the whiskers correspond to the smallest and largest datum within 1.5 IQR.

**Figure 12** shows the distribution histograms of *𝜃_i_* values judged to be closest to the natural tilt of the plane for the observed motion (*n* = 450 for each target tilt *𝜃_b_*), while **Figure 13** shows the corresponding box-and-whisker plots. Data have been pooled across motion durations, repetitions and participants. The median values (IQR) of the responses were 29°45′ (12°30′) and 50°15′ (14°0′) for *𝜃_b_* = 25° and 55°, respectively, significantly different between each other (Kruskal–Wallis, *P* < 0.001). These values indicate that, overall, the smaller target tilt (*𝜃_b_* = 25°) was slightly but significantly (right tailed Wilcoxon signed ranks, *P* < 0.001) overshot by the participants, while the greater target tilt (*𝜃_b_* = 55°) was slightly but significantly (left tailed Wilcoxon signed ranks, *P* < 0.001) undershot. We found no significant effect of repetition for any given *𝜃_b_* and motion duration MD (Kruskal–Wallis, *P* > 0.19), but we found a significant effect of MD on the responses for both values of *𝜃_b_* (*P* < 0.05). Specifically, for both target tilts *𝜃_b_*, the longer the duration of motion, the greater was the median value. However, the magnitude of this effect was relatively small, in as much as the difference between the median values for MD = 0.5 s and those for MD = 0.7 s was equal to 3°30′ for both *𝜃_b_*.

### Conclusion

The results of this experiment confirmed those of the previous experiments, but here the starting condition did not coincide with any of the targets. Moreover, in contrast with the previous experiments, the deviation of the responses from the values consistent with physics was the same at both the low and the high target tilts.

## General Discussion

We showed that observers starting from widely different combinations of slopes and target accelerations can be fairly accurate and precise at finding the specific match that is consistent with physics, that is, they implicitly know Galileo’s law of the inclined plane. Prima facie, our results contrast with the poor performance that has previously been reported for perceptual estimates of naturalness of descending motion along a slope (Bozzi, 1959; Hecht, 1993; Rohrer, 2003). However, those previous studies involved a limited visual context, while we surmise that physically consistent judgments of naturalness of inanimate motion can be arrived at when the visual scene is rich enough to include cues about environmental reference and metric scale or auxiliary cues about such reference are provided by other sensory organs (e.g., vestibular, haptic). Here the 3D visual scene included several cues about the environmental reference and metric scale. The scene was projected at a 1:1 scale, with truthful width-depth rendering, it included high-contrast images of familiar objects and people, with stereo- and perspective cues, textures, directional lights, and shadows. The departure of the present findings from the previous ones reporting poor sensitivity to natural rolling motion with a limited visual context (Hecht, 1993; Rohrer, 2003) is reminiscent of previous studies on interception (Miller et al., 2008) and temporal judgments of gravitational motion (Moscatelli and Lacquaniti, 2011), which found that the performance is much better in the presence than in the absence of a contextual reference in 2D visual scenes.

According to the theory of ecological perception put forth by Gibson (1979), perception is accurate under natural viewing conditions. Our immersive 3D virtual reality is certainly far from being naturalistic, but provides sufficient cues to assess the orientation of objects in space and the effects of gravity, while allowing full experimental control across a range of stimulus conditions (Russo et al., 2017). The projected scene depicted a realistic version of the actual laboratory where the experiments were carried out. It included, in addition to the tilted plane and the rolling ball, human characters, furniture and walls, all drawn with perspective geometry, textures, directional lights, and shadows. Observers viewed the scene stereoscopically at a 1:1 scale, thus with truthful width-depth rendering.

In our animations, the only force acting on the descending ball was gravity, and we assumed negligible viscous friction and rolling resistance. This assumption is legitimate since we previously characterized the motion of a real ball rolling down a smooth incline tilted by 30°, 45°, or 60°, and we found that friction and resistance are indeed negligible (La Scaleia et al., 2014).

In different experiments, we tested two different perceptual abilities, namely the ability to find the slope at which a displayed ball motion looked most natural (slope adjustments, Experiment 1 and 3), and the complementary ability to find the acceleration of the ball that best matched a displayed slope (acceleration adjustments, Experiment 2). Moreover, we used two different methods for slope adjustments, iterative modifications of the slope until satisfied (Experiment 1), and 2-AFC responses with an adaptive staircase (Experiment 3). The results obtained in all experiments were roughly concordant, except that the errors tended to be largest for target tilt equal to 60° in Experiment 1 and for tilt equal to 19° in Experiment 2. In Experiment 3, the errors were comparable for both the low and the high target tilts. Also, the results were generally independent of the travel distance and duration of ball motion, which were randomized across trials.

In Experiments 1 and 2, the participants’ judgments were often closer to those predicted by physics when the trial started from the combination of acceleration and tilt consistent with physics than when it started far away from it. Moreover, the physically congruent starting conditions were judged as natural at the outset more often than the physically incongruent starting conditions, as shown by the higher percentage of immediate responses rendered by the participants without any exploration of the stimuli space. In Experiment 3, the starting condition never coincided with the target.

In the following, we relate our results to those of previous studies. Although a direct comparison is difficult given the idiosyncratic nature of the stimuli and protocols used in each study, nevertheless some inferences can be drawn concerning the potential role of different cues that may concur to the judgments of naturalness of rolling motion along an incline.

### Comparison With Previous Studies on Rolling Motion

As remarked above, a few previous studies investigated the kinematics that observers associate with natural descending motion along an incline, and found a poor adherence to Newtonian mechanics (Bozzi, 1959; Hecht, 1993; Rohrer, 2003). Experiment 1 of Hecht (1993) is the most pertinent to the present results. In this experiment, a wheel with two differently colored halves (so as to make wheel orientation easily noticeable) moved down a ramp (which could have one of two different slopes) at constant speed, constant acceleration (2nd order motion such as that affected by gravity), or monotonically increasing acceleration (4th order motion). Wheel rotation obeyed the same laws as translation, and was paired with it in 9 different combinations (3 translations × 3 rotations). The canonical case was that both translation and rotation obeyed a 2nd order law. Motion was presented on a frontal screen viewed from 4 m distance, and there was no scale factor in the scene.

The results of this study were that events tended to look natural as long as the center of the wheel accelerated, regardless of the exponent of acceleration and regardless of the rotational component. Thus, highly anomalous motions were retained as equally natural as Newtonian motions, and the specific slope of the ramp had no effect on the judgments of naturalness. Moreover, rotation-translation coupling was generally disregarded, consistent with the hypothesis that judgments about dynamic events are primarily based on the particle-motion aspect of the system (Proffitt and Gilden, 1989; Proffitt et al., 1990). In the case of a rolling wheel or ball, the particle motion coincides with the translation of the center of mass, while the extended-body motion is the rotation around the center of mass. Notice that visual contextual cues can be used to derive percepts of rolling objects (Oh and Shiffrar, 2008).

In our experiments, instead, the observers tended to choose the combinations of ball kinematics and incline slope that were consistent with Newtonian mechanics. Moreover, although we did not decouple translation and rotation, the results show that the participants’ judgments took both components of ball motion into account. In fact, had participants responded based on translation only, neglecting rotation, they should have anticipated sliding motions instead of rolling motions. And because sliding acceleration is substantially greater than rolling acceleration (*g*sin*𝜃* instead of 5/7 *g*sin*𝜃*), they should have chosen systematically smaller tilt angles in Experiment 1 (13° instead of 19°, 27° instead of 39°, 38° instead of 60°), and larger accelerations in Experiment 2 (corresponding to 27° instead of 19°, 63° instead of 39°30′, and roughly vertical motion instead of 60°).

One recent study (Masin, 2016) used a real world setting to address the question of whether laypersons can estimate the duration of descent of a ball rolling down an inclined plane placed in a frontal plane (instead of the sagittal plane of the present experiments). A ball was placed at various positions on top of a bar tilted by different angles in a factorial design. Participants assessed descent durations either without seeing or after seeing the ball rolling down the slope. In both cases, the resulting pattern of factorial curves qualitatively corresponded to that implied by Galileo’s law, confirming that the functional relationships defined by this law were perceptually represented. However, participants systematically overestimated both the time of descent of the ball and the inclination of the plane.

### Slant and Acceleration Perception

Whether our participants matched plane slopes to ball kinematics (slope adjustments) or they matched ball kinematics to plane slopes (acceleration adjustments), they had to discriminate implicitly between different slopes as well as between different accelerations. In this respect, the present results can be related indirectly to the vast literature on slant perception and acceleration perception.

With regards to the former, the specific, quantitative results depend on the visual context (mono- versus binocular, texture, etc.), viewing distance (near versus far) and task (e.g., verbal versus motor reports) employed in each study, but most studies agree that the inclination of a plane relative to the horizontal is overestimated, often grossly (Kammann, 1967; Proffitt et al., 1995; Bhalla and Proffitt, 1999; Bridgeman and Hoover, 2008; Allison et al., 2009; Li and Durgin, 2010; Durgin and Li, 2011; Hecht et al., 2014). Thus, Li and Durgin (2010) reported that the actual differences in slant of textured surfaces viewed in 3D virtual reality at 1–2 m viewing distance (comparable to our case), tilted by 6°–24°, are exaggerated in perception by a factor of about 1.5. Hecht et al. (2014) showed wooden ramps tilted by either 15 or 45° in a real world setting to observers placed at 2 m distance, and found overestimates by about 15–25°. As for the precision of slant discrimination, Li and Durgin (2010) reported Weber fractions of about 7%.

In the present experiments, we can consider as theoretical errors the deviations of the judgments from the values predicted by physics. These errors tended to be smaller than those typically reported in slant perception experiments, and consisted in either overshoots or undershoots depending on the target angle. For slope adjustments, the absolute errors of the median judgments ranged from about 1° (for target tilts of 19° and 39° of Experiment 1), to 5° (for 25° and 55° tilts of Experiment 3), up to 13° (for 60° tilt of Experiment 1). For acceleration adjustments (Experiment 2), the equivalent errors in tilt were about 6° (at 39 and 60°) and 14° (at 19°). The lowest target tilts (19° in Experiment 1 and 2, 25° in Experiment 3) tended to be overshot, while the highest tilts (55° in Experiment 3, 60° in Experiment 1 and 2) tended to be undershot. These trends at the extreme values of the target angles might reflect a central tendency effect. Also the precision of the estimates was generally high. In Experiment 1, the precision was about 4° for all target tilts, corresponding to about 6% over the explorable range of plane tilts (6°/70°). In Experiment 2, the precision was about 4° to 7°, that is, 5% to 9% of the range of plane tilts (about 3°/84°) corresponding to the explorable accelerations.

Also the acceleration errors made by our participants tended to be smaller than those reported in several studies dealing with detection or discrimination of random accelerations (Gottsdanker et al., 1961; Calderone and Kaiser, 1989; Werkhoven et al., 1992; Brouwer et al., 2002; Watamaniuk and Heinen, 2003; Mueller and Timney, 2016). Detection thresholds and Weber fractions vary widely across studies, but they are generally very high. For instance, Brouwer et al. (2002) reported that a minimum 25% difference between the initial and final speed is necessary for observers to reliably detect the presence of acceleration. Here, instead, in acceleration adjustments (Experiment 2) we found that both the absolute acceleration errors as well as the precision could be as low as about 6% (for the 60° target).

The task of judging the naturalness of a sphere rolling down an incline requires processing jointly the two variables of slant and acceleration. We suggest that, provided the visual scene includes cues about environmental reference and metric scale, joint processing of slant and acceleration may facilitate their discrimination as compared with the discrimination of each variable separately. Indeed, we seldom see objects in isolation, and seen objects are perceived in relation with each other (Palmer, 1999). Thus, it is well known that the spatial context of a scene can facilitate perception of static and moving objects (DeLucia, 1991; McKee and Smallman, 1998; Distler et al., 2000; Albright and Stoner, 2002; Bar, 2004; Gilroy and Blake, 2004; Miller et al., 2008).

Accurate estimates of natural combinations of slope inclination and ball kinematics may be consistent with Gibson’s (1979) and Proffitt’s (2009) idea that the perception of a surface layout is a perception of affordance, that is, of the relationship between the physical attributes of the perceived object and our potential actions with it. In our experimental conditions, a ball rolling down the incline toward the observer represented a potentially catchable object, and we know that interceptions of such objects is typically quite accurate from the first attempt (La Scaleia et al., 2014, 2015).

### Eye Movements and Optic Cues

Since we did not record eye movements, we do not know whether and how they affected the perceptual responses. In theory, they may have contributed to the judgments of naturalness, since it is known that motions that can be construed as natural events (whether biological or inanimate) are easier to track with eye movements than motions deviating from such natural models (de’Sperati and Viviani, 1997; Souto and Kerzel, 2013). In particular, it has been shown that disks with rotational and translational motion that was congruent with an object rolling on the ground elicited faster eye tracking movements during pursuit initiation than incongruent stimuli, and this behavior was due to visually driven predictions (Souto and Kerzel, 2013). In the present experiments, translation and rotation of the rolling ball were always congruent between each other, but their time profile was either consistent or inconsistent with physics. Thus, it is possible that the ability to track the kinematic profiles consistent with physics better than the other profiles may have influenced the final judgments.

As for the optic cues we considered (rate of change of the visual angle subtended by the ball and of the angular gap with the end position on the incline), in theory they might explain the better performance at larger tilts of the plane than at 19° in Experiment 2, since these signals were potentially larger at higher tilts. However, they cannot explain the opposite results found in Experiment 1, where performance was better at 19° than at higher tilts.

### Neural Simulations of Physical Dynamics

Early studies emphasized the poor ability of humans to apprehend Newtonian mechanics perceptually (Bozzi, 1959; McCloskey and Kohl, 1983). For instance, it was shown that people have difficulties assessing the dynamics of mechanical systems with more than one dynamically relevant parameter (Proffitt and Gilden, 1989). However, recent research suggests that such difficulties, though real, are context-dependent. People judge erroneously very impoverished stimuli, but can demonstrate full capacity to judge about complex environments when provided with appropriate information. Thus, it has been shown that observers control accurately both the timing and the amplitude of muscle activity when preparing to catch balls of different mass falling from variable heights (Lacquaniti and Maioli, 1989; Zago and Lacquaniti, 2005). Also, observers infer correctly the unobservable mass of colliding objects (Sanborn et al., 2013), the stability of a tower of stacked blocks of virtual bricks (Battaglia et al., 2013), as well as the relative masses of the bricks (Hamrick et al., 2016).

These robust and fast inferences in complex natural scenes where crucial information is missing have been explained by assuming that the brain uses approximate, probabilistic simulations of Newtonian mechanics (Battaglia et al., 2013; Sanborn et al., 2013; Lacquaniti et al., 2015; Hamrick et al., 2016). Neural simulations are approximate because they do not solve the equations of motion analytically, but estimate the possible outcomes through learning (Zago et al., 2005, 2008; Battaglia et al., 2013). They are probabilistic due to the uncertainty arising from noisy sensory processes and incomplete prior knowledge of the environment (Zago et al., 2010; White, 2012; Battaglia et al., 2013; Sanborn et al., 2013; Hamrick et al., 2016; Chang and Jazayeri, 2018). As a result of such simulations, physical knowledge can correctly infer objects properties and predict forthcoming changes of physical scenes, but in some cases can lead to systematic deviations of judgments from true physics (McIntyre et al., 2001; Zago et al., 2004; Battaglia et al., 2013; Mijatović et al., 2014).

In the context of the present results, these notions can account for the fact that the combination of slopes and target accelerations consistent with physics was assessed correctly on average, but judgments were considerably variable and were often biased when the exploration started far away from the correct combination.

## Author Contributions

BLS and MZ conceived and designed the research. FC performed the experiments. FC and MZ analyzed the data. MM and AM provided statistical advice and programs. FC, BLS, MR, BC, SG, AM, Ad’A, FL, and MZ interpreted the results. BLS and MZ prepared the Figures. FC, FL, and MZ drafted the manuscript. FC, BLS, MR, BC, SG, MM, AM, Ad’A, FL, and MZ edited and revised the manuscript. FC, BLS, MR, BC, SG, MM, AM, Ad’A, FL, and MZ approved the final version of the manuscript.

## Conflict of Interest Statement

The authors declare that the research was conducted in the absence of any commercial or financial relationships that could be construed as a potential conflict of interest.

## Footnotes

1 The experimental range of plane tilts was compatible with a ball rolling down without slipping or bouncing. This had been verified in previous experiments with a real incline and ball (La Scaleia et al., 2014).

2 http://www.R-project.org/

3 All values are integer, because the discretization of the responses was 1°.

4 A tilt of 39°30′ (instead of 39° as in the previous experiment) was dictated by the size of the acceleration step used in the protocol.

5 These values are non-integer because the discretization of the responses was 4°6′
